# Ruxolitinib Alleviates Uveitis Caused by *Salmonella typhimurium* Endotoxin

**DOI:** 10.3390/microorganisms9071481

**Published:** 2021-07-11

**Authors:** Lin Du, Yolanda Wong Ying Yip, Him Kwan Ng, Bo Man Ho, Jing-Na He, Sun On Chan, Chi Pui Pang, Wai Kit Chu

**Affiliations:** 1 Department of Ophthalmology and Visual Sciences, The Chinese University of Hong Kong, Hong Kong, China; nicki_ovs@link.cuhk.edu.hk (L.D.); yolandayip@cuhk.edu.hk (Y.W.Y.Y.); KwanHimNg@link.cuhk.edu.hk (H.K.N.); bmho@link.cuhk.edu.hk (B.M.H.); jingnahe@cuhk.edu.hk (J.-N.H.); cppang@cuhk.edu.hk (C.P.P.); 2 Molecular Biotechnology Programme, School of Life Sciences, The Chinese University of Hong Kong, Hong Kong, China; 3 School of Biomedical Sciences, The Chinese University of Hong Kong, Hong Kong, China; sunonchan@cuhk.edu.hk

**Keywords:** ruxolitinib, JAK inhibitor, EIU, uveitis, LPS

## Abstract

Uveitis is characterized by inflammatory lesions of intraocular structures. It is one of the important manifestations in patients with Reiter’s syndrome, an inflammatory arthritis, which is caused by enteric infection with bacteria, including Salmonella typhimurium. Corticosteroids remain the most frequently used therapies against uveitis associating with inflammatory arthritis. However, the long-term administration of steroids results in many side effects, and some uveitis patients do not respond to steroid treatment. Non-steroidal treatments are needed for uveitis patients. Our previous study found that Janus kinase (JAK) 1/2 inhibitor, ruxolitinib could suppress the expression of proinflammatory mediators in the ciliary body and iris. However, the impacts of ruxolitinib on ophthalmic features in uveitic eyes are still unknown. In this study, Salmonella typhimurium endotoxin-induced uveitis (EIU) was induced in Sprague Dawley rats by the injection of lipopolysaccharide (LPS). Compared with LPS-induced rats treated with water, ruxolitinib significantly attenuated the clinical manifestations, infiltrating cells and protein exudation in the aqueous humor, and retina–choroid thickening. Amplitudes of b-wave in both scotopic and photopic electroretinogram (ERG), and the amplitude of a-wave in scotopic ERG in EIU animals were alleviated by ruxolitinib. Collectively, we propose ruxolitinib could attenuate endotoxin-induced uveitis and rescue visual functions in rats by inhibiting the JAK2-STAT3 pathway.

## 1. Introduction

Reiter’s syndrome (RS), also called reactive arthritis, is caused by infections with enteric bacteria, including Salmonella, Yersinia and Shigella [1]. It has been reported that around 12% of the RS patients would develop anterior uveitis [2]. As an intraocular inflammatory disease, uveitis contributes to approximately 10–15% of legal blindness and up to 35% of patients exhibiting unilateral or bilateral visual impairment permanently in the developed world [3,4]. 

The rat model of endotoxin-induced uveitis (EIU) is an experimental uveitis model elicited by the systemic injection of Salmonella typhimurium endotoxin lipopolysaccharide (LPS), which leads to acute uveal inflammation, the massive infiltration of cells and protein exudation in anterior segment of the eyes, accompanied with mild posterior inflammation [5]. It is a useful model not only for the investigation of mechanisms in ocular inflammation, but also for the evaluation of the pharmacological efficacy of potential novel drugs [6,7,8,9]. Previous studies identified resident tissue macrophages, CD11b + Ly6C + monocytes, neutrophil, and small numbers of T cells were induced by LPS via the Toll-like receptor 4 (TLR4) and NF-κB pathways [10,11,12,13,14,15]. These immune cells contributed to the pathogenic processes of EIU by producing proinflammatory mediators, including tumor necrosis factor-alpha (TNF-α), interleukin-6 (IL-6), interleukin-1β (IL-1β), monocyte chemoattractant protein-1 (MCP-1) [10,11,12,13,14,15].

Several clinical studies and case reports showed that corticosteroids could be used to treat uveitis associated with RS [16,17]. Nevertheless, some steroid-treated patients may have several intraocular and systemic side effects, such as hepatic toxicity and high intraocular pressure leading to optic neuropathy [18]. In addition, some uveitis is recurrent and difficult to be cured [19]. Therefore, there has been vigorous research in the pathogenesis and search for novel therapies for uveitis. 

Recently, the Janus kinase signal transducer and activator of the transcription (JAK-STAT) signaling pathway has been studied to identify potential anti-inflammatory therapies. This pathway has been reported in inflammatory and autoimmune diseases, including myeloproliferative neoplasms, rheumatoid arthritis, inflammatory bowel diseases, multiple sclerosis, psoriasis and acute lymphoblastic leukemia [20]. The phosphorylation of STATs by JAKs leads to their translocation into the nucleus and transcription of downstream inflammatory factors [21,22,23,24]. Ruxolitinib is an orally administrated, reversible class I inhibitor and competes with ATP in the catalytic site of the JAK 1 and 2 tyrosine kinases. It has been approved in many countries, including the U.S. and EU countries for the treatment of myelofibrosis and polycythemia vera [25]. The concentrations of ruxolitinib in plasma peak within one hour after administration and decline in a monophasic or biphasic manner, with a mean terminal half-life of 2.3 h [26]. Several studies indicate that ruxolitinib is a potential salvage therapy for the corticosteroid-refractory Graft-versus-Host Disease (GvHD) after allogeneic hematopoietic stem cell transplantation (HSCT) [27,28]. However, due to risks of potentially serious adverse effects, including myelosuppression, ruxolitinib should be administrated under close clinical monitoring.

Our previous study has demonstrated that growth hormone-releasing hormone receptor (GHRHR) could activate STAT via binding to JAK2 [11]. This GHRHR-JAK2-STAT3 signaling axis appears to play a key role in endotoxin-induced uveitis. Importantly, inhibiting GHRHR or JAK2 by a GHRHR antagonist or ruxolitinib, respectively, could suppress the expression of proinflammatory mediators in the ciliary body and iris [9]. However, the expression of proinflammatory mediators cannot always reflect the severity of uveitis, as the RNA and proteins can only be extracted from sacrificed animals; thus, important morphological and functional ophthalmic features are not quantified. In this study, we quantified the morphological and functional ophthalmic features, as well as the conventional post-mortem histological features, to demonstrate the anti-inflammatory effects of ruxolitinib in both anterior and posterior segments of EIU-induced uveitic rat eyes.

## 2. Materials and Methods

### 2.1. Induction of EIU and Ruxolitinib Treatments

All animal experiments were conducted in accordance with the guidelines of the Association for Research in Vision and Ophthalmology (ARVO) Statement on Use of Animals in Ophthalmic and Vision Research. Ethics approval for this study was obtained from the Animal Experimentation Ethics Committee of the Chinese University of Hong Kong (protocol code 20-052-HMF issued on 27 April 2020). Female Sprague Dawley rats, weighted 150–200 g, aged 6–8 weeks, were obtained from the Laboratory Animal Service Center of the Chinese University of Hong Kong. The animals were maintained at 25 °C in 12:12 h light–dark cycles, with free access to food and water. Lipopolysaccharide (LPS) from Salmonella typhimurium (Sigma-Aldrich, St. Louis, MO, USA) was dissolved in sterile pyrogen-free phosphate buffer saline (PBS) and EIU was induced by the injection of 0.1 mL of 1 mg/kg LPS solution into one footpad [29]. JAK inhibitor ruxolitinib (ApexBio, Houston, TX, USA) was dissolved in 25 μL of DMSO and then suspended with 475 μL of distilled water within 5 min before feeding intragastrically into rats at dosages of 8 mg/kg and 16 mg/kg. Rats were fed with ruxolitinib intragastrically at 2 and 6 h after LPS injection. Twenty-four hours after LPS injection, rats were sacrificed, and eyeballs were enucleated. The left eyes were fixed in 4% paraformaldehyde for histological analyses and aqueous humor collected from right eyes was used for cell counting and the measurement of protein concentration.

Rats were randomly divided into four group: (i) PBS, rats were mock-induced with PBS into one footpad and fed with water; (ii) LPS + water, rats were induced with LPS and fed with water; (iii) LPS + Low Rux, rats were induced with LPS and fed with 8 mg/kg ruxolitinib at 2 and 4 h after LPS injection; (iv) LPS + High Rux, rats were induced with LPS and fed with 16 mg/kg ruxolitinib at 2 and 4 h after LPS injection. All results were collected from two repeated experiments. Individual data points from both experiments were. 

### 2.2. Clinical Scoring

Animals were examined with a slit lamp (Kowa Product SL-15, Bagshot, UK) and a microscope (Leica M840, Wetzlar, Germany) at baseline and 24 h after LPS injection. Clinical manifestations of EIU were graded from a score of 0 to 4 by a blinded observer, following a previous published criteria [12]: 0 = no inflammatory reaction; 1 = discrete dilation of iris and conjunctival vessels; 2 = moderate dilation of iris and conjunctival vessels with moderate flare in the anterior chamber; 3 = intense iridal hyperemia with intense flare in the anterior chamber; 4 = same clinical signs as 3 with the presence of fibrinoid exudation in the pupillary area and miosis impeding the observation of intraocular inflammatory cells. EIU was considered positive when the clinical score was >1.

### 2.3. Retinal Examination by Confocal Scanning Laser Ophthalmoscope (CSLO) and Optical Coherence Tomography (OCT)

Spectral-domain optical coherence tomography (SD-OCT) and confocal scanning laser ophthalmoscopy (cSLO) (Heidelberg Retina Angiograph 2, Heidelberg Engineering GmbH, Dossenheim, Germany) were used for fundus and retinal imaging. Prior to imaging, rats were anesthetized by the intraperitoneal injection of 75 mg/kg ketamine and 10 mg/kg xylazine. Pupils were dilated with the topical application of mydriatics (0.5% tropicamide and 0.5% phenylephrine). After 2 min, rats were placed on a custom-built platform with the head fixed in marked position for imaging. Sterile artificial tears (Alcon, Inc., Puurs, Belgium) were applied to the cornea every 2 min throughout the imaging process to keep the eyes fully lubricated. The cSLO infrared reflectance was recorded using a light source with a 820 nm wavelength to provide a planar visualization of the retina. The scan rate was 16 frames per second. Eye tracking (a retinal positioning technology widely applied for the exact same retinal location to be ‘‘locked on’’ and scanned) was activated during imaging. A 558 widefield noncontact lens (Heidelberg Engineering GmbH) was installed to the camera for the acquisition of high-quality images in a wider view of the fundus. A total of 15 images at the same retinal location at the same focal depth were scanned and averaged automatically by a built-in software to increase the signal-to-noise ratio. Each image frame represented approximately 40% of the total retinal area [30,31]. Six images covering the whole retinal regions were acquired, with the focus on the retinal ganglion cells layer. 

The OCT system used a superluminescent diode light source with a center wavelength of 870 μm. The OCT parameters were modified to adapt imaging in rats according to the technical advice from the manufacturer (Heidelberg Retina Angiograph 2, Heidelberg Engineering GmbH, Dossenheim, Germany). Thus, infrared retinal fundus photographs and OCT images could be simultaneously captured on the exact retinal focus, which ensured the high quality of OCT imaging in the retina [31]. In each retina, the optic nerve head was centered on a square scanning region covered by 19 OCT cross-sections. Nine images for each B-scan at the same retinal location were captured and averaged [32]. The software from Heidelberg Engineering was used for the quantification of retinal–choroidal thickness (RCT). In the OCT images, 3 concentric circles with diameters of 1 mm, 2 mm and 3 mm were centered on an anchor position marked close to the optic disc automatically by the software in order to maintain the exact same location in follow-up OCT images. The outer 2 concentric circles were divided into 8 grids by 2 lines intersecting perpendicularly. Within each grid, the RCT was measured and averaged. The fold changes of RCT compared with baseline RCT were analyzed and presented as mean ± standard error of mean.

### 2.4. Electroretinography (ERG)

Rats were kept in dark adaption at least 12 h before ERG recording by Diagnosys LLC (Lowell, MA, USA). Rats were anesthetized and pupils were dilated following the same protocol for in vivo imaging. An electrode consisting of gold loop wire was lightly placed on the periphery of the cornea, with the reference electrode placed in the mouth and ground electrode inserted subcutaneously into the hind leg. The electrode–cornea contact was further optimized by moistening the cornea with artificial tear fluid. During ERG measurement, rats were placed on a heated platform to maintain the body temperature at 37 °C in a dark room with only a dim red light. Visual functions were quantified by recording scotopic and photopic ERG with a Diagnosys Espion system and the ColorDome light emitting diode (LED) full-field stimulator (Diagnosys LLC, Lowell, MA, USA). In the scotopic condition, ERGs were recorded under 6 to 10 white-flash stimuli with intensities ranging 0.001, 0.01, 0.1, 1, and 10 cd.s/m^2^. Photopic ERGs were recorded under 6 to 10 flash stimuli at each light intensity ranging from 0.5, 1, 5, 10, and 30 cd.s/m^2^. The amplitudes of b-wave were measured from trough of a-wave to the peak of the b-wave. The amplitudes of a-wave and b-wave were averaged in each intensity for comparison. 

### 2.5. Cell Counting and Protein Quantification in Aqueous Humor

A 30-gauge needle was inserted into the anterior chamber with caution to avoid puncturing the lens. All the aqueous humor samples were kept on ice during cell counting and protein quantification. Aqueous humor was diluted ten times in PBS and then stained with Trypan-blue. Live cells were counted using a hemocytometer under a light microscope (Leica Microsystems, Wetzlar, Germany). The rest of aqueous humor sample was centrifuged at 13,000× *g* for 15 min at 4 °C. Protein concentrations of the supernatant were then measured using a total protein assay kit (Beyotime, Shanghai, China).

### 2.6. Histopathological Evaluation

Rats were sacrificed under deep anesthesia and eyes were collected and briefly rinsed with cold PBS, followed by immersion in 4% paraformaldehyde for 24 h at 4 °C. After dehydration, they were embedded in paraffin and serial sagittal sections (5 μm thick) were cut through the pupil–optic nerve position and stained with hematoxylin and eosin (H&E). The anterior chamber, iris–ciliary body, vitreous, and retina were examined under a light microscope (DMRB, Leica Microsystems, Wetzlar, Germany). 

### 2.7. Statistical Analysis

All results were collected from two independent experiments. All individual data were analyzed to obtain the mean ± standard error of mean. Comparisons were statistically analyzed using Mann–Whitney tests by using the SPSS software version 19.0 (IBM, Chicago, IL, USA) and the GraphPad Prism 8.0 (GraphPad Software, Inc., La Jolla, CA, USA) software. *p* values less than or equal to 0.05 are considered as statistically significant.

## 3. Results

### 3.1. Ruxolitinib Alleviated Clinical Manifestations of Inflammation in Eyes 

**Figure 1:**
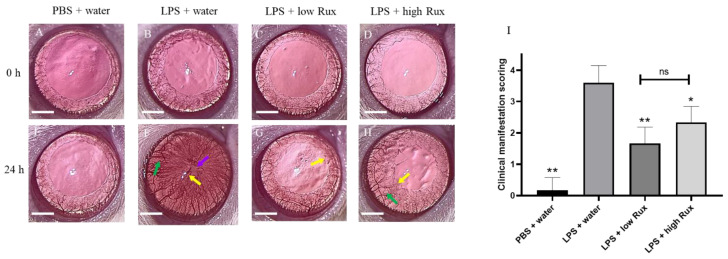
Clinical manifestations of ocular inflammation in rat eyes. Eyes were examined by slit lamp and microscope at baseline (0 h) and 24 h after LPS injection. (**A**–**D**) No ocular inflammation was observed in all eyes at baseline. (**E**) No ocular inflammation was observed in the PBS induced eyes 24 h after PBS injection. (**F**) Severe ocular inflammations, including hyperemia (green arrow), edema (yellow arrow) and synachesia (purple arrow), were observed in the iris of LPS-treated rats 24 h after EIU induction. (**G**,**H**) The ocular inflammation in iris was alleviated in rats treated with 8 mg/kg (low Rux) and 16 mg/kg ruxolitinib (high Rux). (**I**) Quantification of the clinical scores of ocular inflammations in rats. A total of 6 eyes from 6 individual rats were analyzed in each group. Data are shown as mean ± standard error of mean (SEM). Statistics are evaluated by Mann–Whitney U test. * and ** represent *p* < 0.05 and *p* < 0.01, respectively, when compared to the LPS + water group. ns represents no significant difference. Scale bar: 2 mm.

To evaluate whether ruxolitinib exerts anti-inflammatory effects in the anterior segment of the eye after the induction of EIU, the eyes were examined using a slit lamp and microscopy at baseline and 24 h after LPS injection. As shown in Figure 1A–D, eyes at baseline showed clear blood vessels and smooth iris frill. Twenty-four hours after LPS injection, severe ocular inflammation indicated by the presence of hyperemia, edema and synachesia were observed in the iris (Figure 1F), whereas no inflammatory features were found in rats with PBS injection (Figure 1E). These inflammatory features were significantly alleviated in EIU rats treated with ruxolitinib at both low (8 mg/kg) and high doses (16 mg/kg) (Figure 1G,H). The quantitative evaluation of these clinical scores showed a significant reduction in animals with the oral administration of ruxolitinib at low dose (*p* < 0.01) and high dose (*p* < 0.05) when compared with animals fed with water after LPS injection (Figure 1I).

### 3.2. Ruxolitinib Reduced Retinal–Choroidal Thickness and Infiltrating Cells in the Vitreous and Retina in EIU

**Figure 2:**
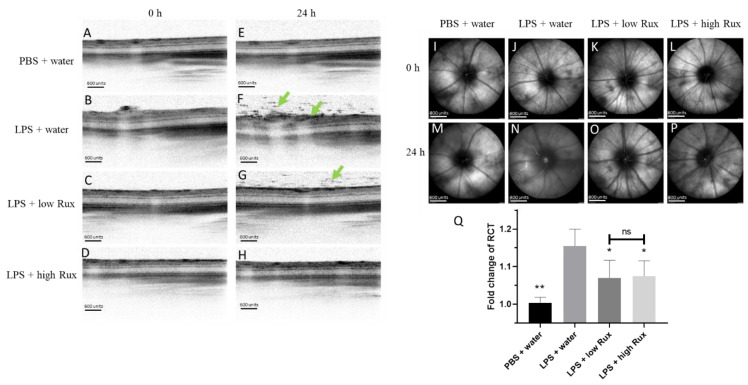
Ocular inflammation examined by cSLO and SD-OCT in posterior segment of rat eyes. No detectable inflammation features were found in SD-OCT images (**A**–**D**) and cSLO images (**I**–**L**) from all four groups of rats at baseline (0 h). Twenty-four hours after EIU induction, lots of dark signals (green arrows) were detected in vitreous and retina in SD-OCT image (**F**) and cSLO image (**N**) in LPS-injected animals treated with water, compared with no obvious inflammation in rats with PBS mock induction (**E**,**M**). The inflammation features were alleviated with treatment of 8 mg/kg (low Rux, **G**,**O**) and 16 mg/kg ruxolitinib (high Rux, **H**,**P**). (**Q**) The fold change of RCT in rat eyes between the baseline and 24 h after PBS or LPS injection. A total of 6 eyes from 6 individual rats were analyzed in each group. Data are shown as mean ± standard error of mean (SEM). Statistics are evaluated by Mann–Whitney U test. * and ** represent *p* < 0.05 and *p* < 0.01, respectively, when compared to the LPS + water group. Ns represents no significant difference. Scale bars: (**A**–**H**): 600 units, (**I**–**P**): 800 units.

We further investigated whether ruxolitinib could ameliorate ocular inflammation developed in the posterior segment of the eye in EIU rats. Eyes were examined by confocal scanning laser ophthalmoscopy (cSLO) and spectral-domain optical coherence tomography (SD-OCT) at baseline and 24 h after LPS injection. Retinal layers and vessels could be clearly identified at baseline (Figure 2A–D). Twenty-four hours after LPS induction, lots of dark signals were detected in the vitreous, indicating infiltrating cells in the vitreous (Figure 2E–H). In addition, clear cSLO images could be detected at baseline (Figure 2I–L) while a blurred fundus image was observed 24 h after LPS induction (Figure 2M,N), probably due to the accumulation of infiltrating cells. With the treatment of low and high dosages of ruxolitinib, the fundus images were better defined (Figure 2O,P). Furthermore, measurements of the retinal–choroidal thickness (RCT) on the OCT images showed that there was a significant increase in retinal thickness (*p* < 0.01) after LPS induction (Figure 2Q). The fold change of RCT in EIU rats treated with low and high dosages of ruxolitinib were reduced significantly (*p* < 0.05, respectively) (Figure 2Q). In conclusion, ruxolitinib was able to alleviate ocular inflammation in posterior segments of eyes in EIU rats.

### 3.3. Electroretinography

**Figure 3:**
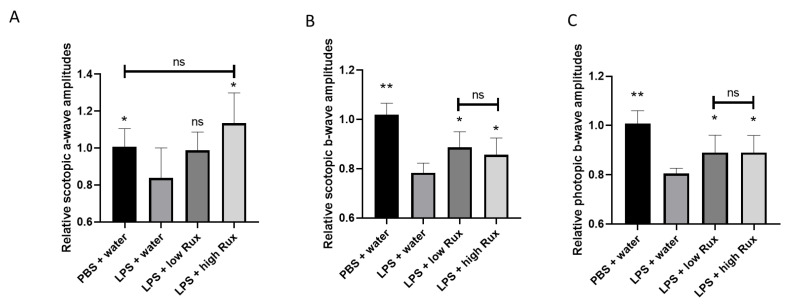
ERG amplitudes were recovered in EIU-induced rats treated with ruxolitinib. (**A**) The amplitude of a-wave in scotopic ERG were significantly reduced under light intensity of 0.001 cd.s/m^2^. Scotopic (**B**) and photopic (**C**) b-waves were recorded under light intensities of 10 cd.s/m^2^ and 30 cd.s/m^2^, respectively. In all ERG comparisons, the ERG amplitudes were reduced significantly 24 h after LPS injection compared to the PBS mock induction. The reduced ERG amplitudes were significantly elevated after treatment with 8 mg/kg (low Rux) and 16 mg/kg ruxolitinib (high Rux). In Figure 3A, 5 eyes from 5 individual rats were analyzed in each group. In Figure 3B,C, 6 eyes from 6 individual rats were analyzed in each group. Data are shown as mean ± standard error of mean (SEM). Statistics are evaluated by Mann–Whitney U test. * and ** represent *p* < 0.05 and *p* < 0.01, respectively, when compared to the LPS + water group. ns represents no significant difference.

Apart from the morphological observations, the inflammatory influences on visual functions were further assessed by photopic and scotopic ERG. In scotopic ERG, a significant reduction in the amplitudes of a-wave was observed after LPS induction (Figure 3A), suggesting rod cell functions were inhibited. The reduced amplitude of a-wave could be significantly (*p* < 0.05) elevated by treatment of a high dosage of ruxolitinib in EIU rats (Figure 3A). In both photopic and scotopic ERG, the amplitudes of b-wave were reduced significantly (*p* < 0.05) after LPS induction, indicating impairments of functions of retinal interneurons under both light adaptation and dark adaptation (Figure 3B,C). Both the diminished scotopic and photopic b-wave amplitudes were significantly recovered in rats treated with low and high dosages of ruxolitinib (*p* < 0.05, respectively) (Figure 3B,C), suggesting ruxolitinib was able to rescue the impaired visual functions in EIU rats.

### 3.4. Ruxolitinib Alleviated Protein Secretion into Aqueous Humor and Infiltrating Cells in Anterior and Posterior Segments 

**Figure 4:**
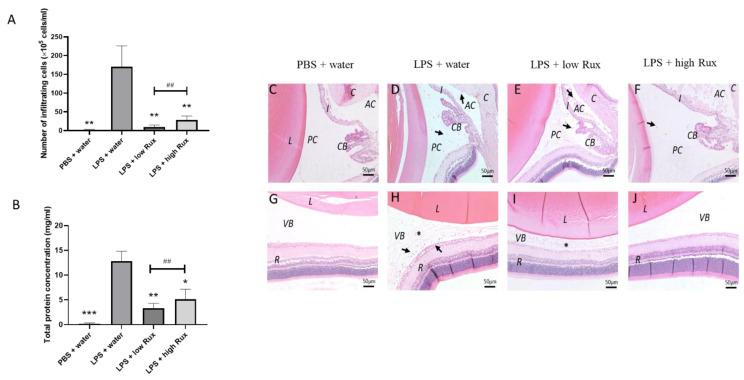
The effects of ruxolitinib on cellular infiltration and protein secretion into the aqueous humor. (**A**) The number of infiltrating cells in aqueous humor were counted. (**B**) Total protein concentration of in aqueous humor were quantified. 6 eyes from 6 individual rats were analyzed in each group. Data are shown as mean ± standard error of mean (SEM). Statistics are evaluated by Mann–Whitney U test. * and ** represent *p* < 0.05 and *p* < 0.01, respectively, when compared to the LPS + water group. ## represents *p* < 0.01 between the LPS + low Rux and LPS + high Rux groups. ns represents no significant difference. Histology sections of the anterior (**C**–**F**) and posterior segments (**G**–**J**) 24 h after PBS or LPS injection are shown. (**C**,**G**) showed that no obvious infiltrating cells and protein secretion were observed in anterior chamber, posterior chamber and vitreous after PBS mock induction. In (**D**,**H**), infiltrating cells (arrows) and protein secretion (asterisks) were detected in anterior chamber, posterior chamber and vitreous in LPS-induced rats treated with water. (**E**,**I**) In rats treated with 8 mg/kg (low Rux), infiltrating cells were slightly reduced in both anterior chamber, posterior chamber and vitreous. (**F**,**J**) In rats treated with 16 mg/kg (high Rux), most of the infiltrating cells and secreted protein were reduced in anterior chamber, posterior chamber and vitreous. L: lens, AC: anterior chamber, PC: posterior chamber, I: iris, CB: ciliary body, VB: vitreous body, R: retina. Scale bar: 50 μm.

The injection of LPS-induced substantial accumulation of infiltrating cells in the posterior segment of the eyes as detected in SD-OCT results (Figure 2F). To directly quantify the intraocular inflammation, infiltrating cells and protein concentration were measured in the aqueous humor. As showed in Figure 4A, no obvious infiltrating cells were detectable in PBS-induced rats, whereas a significant increased number of cells was observed in LPS-induced rats (*p* < 0.01). The number of infiltrating cells was significantly reduced by ruxolitinib treatment at both low and high dosages (*p* < 0.01, respectively). The concentration of proteineous substances in aqueous humor from LPS-induced rats was much higher (*p* < 0.001) than the PBS-induced rats. Additionally, the elevated aqueous protein levels were significantly reduced in rats treated with ruxolitinib at low (*p* < 0.01) and high dosages (*p* < 0.01) (Figure 4B). Thus, our results suggested that ruxolitinib treatments could prevent the EIU-induced infiltration of inflammatory cells and the release of inflammatory proteins in aqueous humor.

Histology examination also showed consistent results with the live measurements of cSLO and SD-OCT. Histopathological changes, infiltrating cells and protein accumulation were observed in anterior segment and vitreous 24 h after EIU induction (Figure 4D,H) in contrast to the PBS-induced rats (Figure 4C,G). A reduced cell infiltration was observed in rats treated with ruxolitinib at both low and high dosages (Figure 4E,F,I,J). In addition, the accumulation of protein was not detectable in anterior chamber and vitreous in rats treated with high-dose ruxolitinib (Figure 4F,J).

## 4. Discussion

In this study, we investigated the morphological, functional and histological ophthalmic features to evaluate the anti-inflammatory effects of ruxolitinib in a rat model of EIU. In this study, female rats were studied, as uveitis with systemic involvement has been reported to be predominant in females [33]. We found that ruxolitinib could effectively attenuate ocular inflammation induced by LPS in both anterior and posterior segments of the eye. The treatment of ruxolitinib alleviated clinical manifestations in EIU rats. It also reduced cellular infiltration and protein secretion into the aqueous humor and vitreous. A previous study found that the JAK-STAT pathway was required for the development of Th17 cells in an autoimmune uveitis animal model [34]. Future studies will be needed to identify the target cell types affected by ruxolitinib. In addition, the increased retinal–choroidal thickness and impaired visual function caused by EIU induction were ameliorated by the treatment of ruxolitinib. Future studies will be needed to distinguish whether the improved ERG effects are caused by the reduced blockage of the optical path, as shown by less infiltrating cells and protein secretion, or by genuine protective effects on the photoreceptors and retinal interneurons. Together, our findings showed that ruxolitinib is able to suppress intraocular inflammation.

In our previous study, by studying the growth hormone-releasing hormone receptor (GHRHR) in LPS-induced intraocular inflammation, we identified a mechanism that GHRHR and JAK2 are expressed by the ciliary and iris epithelial cells and can bind to each other [11]. After the induction of EIU, NF-κB subunit p65 is phosphorylated in response to lipopolysaccharide (LPS) stimulation, which would lead to the transcriptional up-regulation of growth hormone-releasing hormone receptor (GHRHR). GHRHR could directly bind to JAK2, which enhanced the phosphorylation of STAT3 and the expression of proinflammatory factors. We found this STAT3 phosphorylation could be inhibited by the GHRHR antagonist or ruxolitinib [11]. Furthermore, ruxolitinib was able to suppress the expression of proinflammatory factors, including COX2, IL-17A, MMP9, iNOS and IL-6 induced by EIU induction [11]. In addition to cytokine expression, it is also important to quantify the ocular inflammatory status in live EIU rats after treating ruxolitinib. Our current study quantified in vivo effects of ruxolitinib on retinal structures and functions, which are important for the future development of JAK inhibitors in treating uveitis.

Several case reports have shown the effects of JAK inhibitors in resolving uveitis. Miserocchi et al. reported oral JAK inhibitors baricitinib and tofacitinib were able to reduce intraocular inflammation in four patients suffering from juvenile idiopathic arthritis (JIA) and associated uveitis [35]. Another case report also described the successful treatment of a 22-year-old woman suffering from JIA associated with active uveitis and macular edema in both eyes by oral tofacitinib [36]. There was no placebo group for systematic comparisons and statistical analyses in these case reports. Our animal study can provide a scientific basis to justify the use of JAK inhibitors in treating uveitis. Nevertheless, it is important to notice that all five of these patients in both case reports originally showed no or partial responses to biologic treatments, including rituximab (targeting CD20), tocilizumab (targeting IL-6 receptor) and TNF-alpha blockers such as adalimumab and infliximab. Our results demonstrated that ruxolitinib is a potentially promising treatment against uveitis. It might potentially alleviate severe intraocular inflammation caused by the inflammatory cytokine storm in patients with an acute stage of uveitis resistant to corticosteroid treatment. However, another study reported that drug-related effects, including anemia and thrombocytopenia, could be induced in myelofibrosis patients receiving ruxolitinib [37]. Furthermore, several case reports found that patients receiving ruxolitinib developed pulmonary and extrapulmonary tuberculosis, pneumocystis jiroveci pneumonia, hepatitis B virus reactivation and toxoplasmosis retinitis [38,39,40,41]. Due to concerns regarding these adverse events, the combination of ruxolitinib with existing treatments or the topical ocular administration of ruxolitinib might be an attractive option. A previous study has proven that twice daily topical administrations of 1.5% ruxolitinib cream, although not being used as eye drop, could retain its effectiveness in treating facial vitiligo patients with minor side effects [42]. Moreover, applying 0.003% of another JAK inhibitor, tofacitinib, twice daily on the ocular surface in mice could reduce the expression of proinflammatory factors, including TNF-α, IL-17A and IL-23 [43]. These studies could lead to a hypothesis that topical administration of ruxolitinib eye drop could be a safer treatment in uveitis patients. Although combination treatment or topical ocular treatment might be a safer option, close clinical monitoring of patients should still be included in future trials of applying ruxolitinib in treating uveitis. In addition to treating uveitis, ruxolitinib was reported to protect mice from LPS-induced sepsis by suppressing nitric oxide production and the expression of iNOS, TNF-α and IL-6 [44]. Furthermore, other JAK inhibitors, including tofacitinib, baricitinib and filgotinib, have been reported to be able to alleviate uveitis refractory to topical steroids in several case reports [45]. In addition, our previous study identified the signaling pathway upstream of JAK involves Toll-like receptor 4 (TLR4), a pattern recognition receptor that can be activated by LPS [11]. Through these signaling pathways, downstream proinflammatory mediators, including TNF-α, interleukins, adhesion molecules and chemokines, were released, which could modulate polymorphonuclear leukocyte adhesion, endothelial cell apoptosis and microvasculature in the EIU model [46]. Therefore, targeting TLR4 could be explored to suppress the production of proinflammatory cytokines in LPS-induced uveitis. TLR4 antagonists, including eritoran, have been shown to be effective in treating LPS-induced ocular inflammation [47]. Future studies are needed to evaluate these alternative drug candidates in treating LPS-induced uveitis and their safety profiles.

## Data Availability

The data presented in this study are available within this article.

## References

[1] Hill Gaston J.S., Lillicrap M.S. (2003). Arthritis associated with enteric infection. Best Pract. Res. Clin. Rheumatol..

[2] Lee D.A., Barker S.M., Su W.P., Allen G.L., Liesegang T.J., Ilstrup D.M. (1986). The clinical diagnosis of Reiter’s syndrome. Ophthalmic and nonophthalmic aspects. Ophthalmology.

[3] Rothova A., Suttorp-van Schulten M.S., Frits Treffers W., Kijlstra A. (1996). Causes and frequency of blindness in patients with intraocular inflammatory disease. Br. J. Ophthalmol..

[4] Hsu Y.R., Huang J.C., Tao Y., Kaburaki T., Lee C.S., Lin T.C., Hsu C.C., Chiou S.H., Hwang D.K. (2019). Noninfectious uveitis in the Asia-Pacific region. Eye.

[5] Qin Y.J., Chu K.O., Yip Y.W., Li W.Y., Yang Y.P., Chan K.P., Ren J.L., Chan S.O., Pang C.P. (2014). Green tea extract treatment alleviates ocular inflammation in a rat model of endotoxin-induced uveitis. PLoS ONE.

[6] Tang W., Ma J., Gu R., Ding X., Lei B., Wang X., Zhuang H., Xu G. (2018). Lipocalin 2 Suppresses Ocular Inflammation by Inhibiting the Activation of NF-kappabeta Pathway in Endotoxin-Induced Uveitis. Cell. Physiol. Biochem..

[7] Uchida T., Honjo M., Yamagishi R., Aihara M. (2017). The Anti-Inflammatory Effect of Ripasudil (K-115), a Rho Kinase (ROCK) Inhibitor, on Endotoxin-Induced Uveitis in Rats. Invest. Ophthalmol. Vis. Sci..

[8] Wang X.Q., Liu H.L., Wang G.B., Wu P.F., Yan T., Xie J., Tang Y., Sun L.K., Li C. (2011). Effect of artesunate on endotoxin-induced uveitis in rats. Invest. Ophthalmol. Vis. Sci..

[9] Smith J.R., Hart P.H., Williams K.A. (1998). Basic pathogenic mechanisms operating in experimental models of acute anterior uveitis. Immunol. Cell Biol..

[10] Ren J.L., Yu Q.X., Ma D., Liang W.C., Leung P.Y., Ng T.K., Chu W.K., Schally A.V., Pang C.P., Chan S.O. (2019). Growth hormone-releasing hormone receptor mediates cytokine production in ciliary and iris epithelial cells during LPS-induced ocular inflammation. Exp. Eye Res..

[11] Liang W.C., Ren J.L., Yu Q.X., Li J., Ng T.K., Chu W.K., Qin Y.J., Chu K.O., Schally A.V., Pang C.P. (2020). Signaling mechanisms of growth hormone-releasing hormone receptor in LPS-induced acute ocular inflammation. Proc. Natl. Acad. Sci. USA.

[12] Pouvreau I., Zech J.C., Thillaye-Goldenberg B., Naud M.C., Van Rooijen N., de Kozak Y. (1998). Effect of macrophage depletion by liposomes containing dichloromethylene-diphosphonate on endotoxin-induced uveitis. J. Neuroimmunol..

[13] Wu T.T., Chen T.L., Chen R.M. (2009). Lipopolysaccharide triggers macrophage activation of inflammatory cytokine expression, chemotaxis, phagocytosis, and oxidative ability via a toll-like receptor 4-dependent pathway: Validated by RNA interference. Toxicol. Lett..

[14] McMenamin P.G., Crewe J. (1995). Endotoxin-induced uveitis. Kinetics and phenotype of the inflammatory cell infiltrate and the response of the resident tissue macrophages and dendritic cells in the iris and ciliary body. Invest. Ophthalmol. Vis. Sci..

[15] Chu C.J., Gardner P.J., Copland D.A., Liyanage S.E., Gonzalez-Cordero A., Kleine Holthaus S.M., Luhmann U.F., Smith A.J., Ali R.R., Dick A.D. (2016). Multimodal analysis of ocular inflammation using the endotoxin-induced uveitis mouse model. Dis. Models Mech..

[16] Kiss S., Letko E., Qamruddin S., Baltatzis S., Foster C.S. (2003). Long-term progression, prognosis, and treatment of patients with recurrent ocular manifestations of Reiter’s syndrome. Ophthalmology.

[17] Fischel J.D., Lipton J. (1996). Acute anterior uveitis in juvenile Reiter’s syndrome. Clin. Rheumatol..

[18] Farkouh A., Frigo P., Czejka M. (2016). Systemic side effects of eye drops: A pharmacokinetic perspective. Clin. Ophthalmol..

[19] Agarwal A., Aggarwal K., Gupta V. (2019). Infectious uveitis: An Asian perspective. Eye.

[20] Villarino A.V., Kanno Y., O’Shea J.J. (2017). Mechanisms and consequences of Jak-STAT signaling in the immune system. Nat. Immunol..

[21] Kisseleva T., Bhattacharya S., Braunstein J., Schindler C.W. (2002). Signaling through the JAK/STAT pathway, recent advances and future challenges. Gene.

[22] Rodig S.J., Meraz M.A., White J.M., Lampe P.A., Riley J.K., Arthur C.D., King K.L., Sheehan K.C., Yin L., Pennica D. (1998). Disruption of the Jak1 gene demonstrates obligatory and nonredundant roles of the Jaks in cytokine-induced biologic responses. Cell.

[23] Jamilloux Y., El Jammal T., Vuitton L., Gerfaud-Valentin M., Kerever S., Seve P. (2019). JAK inhibitors for the treatment of autoimmune and inflammatory diseases. Autoimmun. Rev..

[24] Choy E.H. (2019). Clinical significance of Janus Kinase inhibitor selectivity. Rheumatology.

[25] Vainchenker W., Leroy E., Gilles L., Marty C., Plo I., Constantinescu S.N. (2018). JAK inhibitors for the treatment of myeloproliferative neoplasms and other disorders. F1000Research.

[26] Shilling A.D., Nedza F.M., Emm T., Diamond S., McKeever E., Punwani N., Williams W., Arvanitis A., Galya L.G., Li M. (2010). Metabolism, excretion, and pharmacokinetics of [14C]INCB018424, a selective Janus tyrosine kinase 1/2 inhibitor, in humans. Drug Metab. Dispos..

[27] Spoerl S., Mathew N.R., Bscheider M., Schmitt-Graeff A., Chen S., Mueller T., Verbeek M., Fischer J., Otten V., Schmickl M. (2014). Activity of therapeutic JAK 1/2 blockade in graft-versus-host disease. Blood.

[28] Zeiser R., Burchert A., Lengerke C., Verbeek M., Maas-Bauer K., Metzelder S.K., Spoerl S., Ditschkowski M., Ecsedi M., Sockel K. (2015). Ruxolitinib in corticosteroid-refractory graft-versus-host disease after allogeneic stem cell transplantation: A multicenter survey. Leukemia.

[29] Ruiz-Moreno J.M., Thillaye B., de Kozak Y. (1992). Retino-choroidal changes in endotoxin-induced uveitis in the rat. Ophthalmic Res..

[30] Liu S., Li Z.W., Weinreb R.N., Xu G., Lindsey J.D., Ye C., Yung W.H., Pang C.P., Lam D.S., Leung C.K. (2012). Tracking retinal microgliosis in models of retinal ganglion cell damage. Invest. Ophthalmol. Vis. Sci..

[31] Li J., Ren J., Yip Y.W.Y., Zhang X., Chu K.O., Ng T.K., Chan S.O., Pang C.P., Chu W.K. (2017). Quantitative Characterization of Autoimmune Uveoretinitis in an Experimental Mouse Model. Invest. Ophthalmol. Vis. Sci..

[32] Guo L., Normando E.M., Nizari S., Lara D., Cordeiro M.F. (2010). Tracking longitudinal retinal changes in experimental ocular hypertension using the cSLO and spectral domain-OCT. Invest. Ophthalmol. Vis. Sci..

[33] Yeung I.Y., Popp N.A., Chan C.C. (2015). The role of sex in uveitis and ocular inflammation. Int. Ophthalmol. Clin..

[34] Liu X., Lee Y.S., Yu C.R., Egwuagu C.E. (2008). Loss of STAT3 in CD4+ T cells prevents development of experimental autoimmune diseases. J. Immunol..

[35] Miserocchi E., Giuffre C., Cornalba M., Pontikaki I., Cimaz R. (2020). JAK inhibitors in refractory juvenile idiopathic arthritis-associated uveitis. Clin. Rheumatol..

[36] Bauermann P., Heiligenhaus A., Heinz C. (2019). Effect of Janus Kinase Inhibitor Treatment on Anterior Uveitis and Associated Macular Edema in an Adult Patient with Juvenile Idiopathic Arthritis. Ocul. Immunol. Inflamm..

[37] Verstovsek S., Mesa R.A., Gotlib J., Levy R.S., Gupta V., DiPersio J.F., Catalano J.V., Deininger M., Miller C., Silver R.T. (2012). A double-blind, placebo-controlled trial of ruxolitinib for myelofibrosis. N. Engl. J. Med..

[38] Chen Y.H., Lee C.H., Pei S.N. (2015). Pulmonary tuberculosis reactivation following ruxolitinib treatment in a patient with primary myelofibrosis. Leuk. Lymphoma.

[39] Hopman R.K., Lawrence S.J., Oh S.T. (2014). Disseminated tuberculosis associated with ruxolitinib. Leukemia.

[40] Lee S.C., Feenstra J., Georghiou P.R. (2014). Pneumocystis jiroveci pneumonitis complicating ruxolitinib therapy. BMJ Case Rep..

[41] Goldberg R.A., Reichel E., Oshry L.J. (2013). Bilateral toxoplasmosis retinitis associated with ruxolitinib. N. Engl. J. Med..

[42] Rothstein B., Joshipura D., Saraiya A., Abdat R., Ashkar H., Turkowski Y., Sheth V., Huang V., Au S.C., Kachuk C. (2017). Treatment of vitiligo with the topical Janus kinase inhibitor ruxolitinib. J. Am. Acad. Dermatol..

[43] Stevenson W., Sadrai Z., Hua J., Kodati S., Huang J.F., Chauhan S.K., Dana R. (2014). Effects of topical Janus kinase inhibition on ocular surface inflammation and immunity. Cornea.

[44] Li L., He X., Wang X., Sun Y., Wu G., Fang H., Wang C., Luo P., Xia Z. (2020). Ruxolitinib protects lipopolysaccharide (LPS)-induced sepsis through inhibition of nitric oxide production in mice. Ann. Transl. Med..

[45] Spinelli F.R., Meylan F., O’Shea J.J., Gadina M. (2021). JAK inhibitors: Ten years after. Eur. J. Immunol..

[46] Koizumi K., Poulaki V., Doehmen S., Welsandt G., Radetzky S., Lappas A., Kociok N., Kirchhof B., Joussen A.M. (2003). Contribution of TNF-alpha to leukocyte adhesion, vascular leakage, and apoptotic cell death in endotoxin-induced uveitis in vivo. Investig. Ophthalmol. Vis. Sci..

[47] Ekici F., Karaca E.E., Korkmaz S., Yuksel O., Gulbahar O., Alper M., Ercan S., Or M. (2014). Effect of the Toll-like receptor 4 antagonist eritoran on retinochoroidal inflammatory damage in a rat model of endotoxin-induced inflammation. Mediat. Inflamm..

